# Molecular features of IGHV3-53-encoded antibodies elicited by SARS-CoV-2

**DOI:** 10.1038/s41392-020-00287-4

**Published:** 2020-08-25

**Authors:** Francesca Fagiani, Michele Catanzaro, Cristina Lanni

**Affiliations:** 1 Department of Drug Sciences (Pharmacology Section), University of Pavia, V.le Taramelli 14, 27100 Pavia, Italy; 2 Scuola Universitaria Superiore IUSS Pavia, P.zza Vittoria, 15, 27100 Pavia, Italy

**Keywords:** Structural biology, Infectious diseases

An elegant paper by Yuan et al., recently published in Science, provides novel insights into the molecular features of neutralizing antibody responses to the severe acute respiratory syndrome coronavirus 2 (SARS-CoV-2).^1^

According to the principles of the “reverse vaccinology 2.0” postulated by Burton et al.,^2^ the authors explore the interactions between potent neutralizing antibodies from naturally infected donors and their target epitopes, providing key information about structural motifs and binding mode that may facilitate the design of vaccine antigens capable to elicit the immune response against SARS-CoV-2. The vast majority of anti-CoV neutralizing antibodies have been found to specifically target the receptor-binding domain (RBD) of the viral spike (S) protein, thus hindering SARS-CoV-2 binding to the host angiotensin converting enzyme 2 (ACE2) receptor and viral entry.^3^

Yuan and collaborators analyzed 294 anti-SARS-CoV-2 antibodies from COVID-19 patients and demonstrated that among these antibodies the immunoglobulin heavy variable 3-53 (IGHV3-53) represents the most frequently used IGHV gene, with 10% encoded by IGHV3-53. In the cohort investigated by Yuan et al., IGHV3-53 antibodies have been reported to be more potent compared to other germlines, as well as to display lower somatic mutation rates. The authors determined the crystal structures of two antibodies, CC12.1 and CC12.3, encoded by a common IGHV3-53 gene, but belonging to different clonotypes, in order to define the structural features, and to add favorable properties for RBD recognition to IGHV3-53. Notably, among the antibodies tested against live replicating SARS-CoV-2 and pseudovirus, CC12.1 and CC12.3 (IC_50_ ~ 20 ng/mL), isolated from COVID-19 patients, are among the top four highly potent neutralizing antibodies, with a binding affinity (K_d_) of Fabs CC12.1 and CC12.3 to SARS-CoV-2 RBD of 17 and 14 nM, respectively.^1,4^ By performing competitions experiments, Yuan et al. demonstrated that both CC12.1 and CC12.3 bind to the ACE2 binding site on SARS-CoV-2 RBD with an identical angle of approach. Among 17 ACE2 binding residues on RBD, 15 and 11 are within the epitopes of CC12.1 and CC12.3, respectively. Remarkably, several epitope residues are not conserved between SARS-CoV-2 and SARS-CoV, thus explaining, at least in part, the absence of antibody cross-reactivity between these two CoVs.^5^ Such evidence is consistent with data, reported by Ju et al, showing the lack of antibody cross-reactivity with RBDs not only from SARS-CoV, but also from middle east respiratory syndrome coronavirus (MERS-CoV), thus suggesting that SARS-CoV, SARS-CoV-2, and MERS-CoV are immunologically distinct.^5^ As an example, despite SARS-CoV-2 and SARS-CoV display both sequential and structural similarities, diverse viral species-specific responses have been observed in patients.^5^ Such evidence justifies the failures of the attempts to neutralize SARS-CoV-2 by using previously isolated SARS-CoV antibodies.^5^

Moreover, the authors provided evidence that CC12.1 presents immunoglobulin kappa variable1-9 (IGKV1-9) and CCL12.3 IGKV3-20, thereby suggesting that IGHV3-53 can pair with different light chains. Such finding indicates that the identity of the heavy chain, instead of that of the light-chain, might be critical for targeting ACE2 binding site in SARS-CoV-2 RBD.

Furthermore, the complementarity-determining regions (CDRs) of IGHV3-53 were structurally analyzed. Based on structural analysis, the presence of two structural motifs, the NY motif in the CDR H1 and an SGGS motif in the CDR H2, as well as the short length of CDR H3, appear fundamental for the binding to the RBD. CDR H1 and H2 of CC12.1 and CC12.3 antibodies have been found to stabilize the CDR conformation with the surrounding framework and to establish hydrogen bonds with the carbonyl backbone of key amino acids in the RBD. While high similarity in the interaction modes between SARS-CoV-2 RBD and CDR H1 and H2 loops has been found, significant differences in the CDR H3 sequence and conformations have been observed when comparing two antibodies. As an example, while CDR H3 of CC12.2 has been found to establish a hydrogen bond with RDB Y453, CDR H3 of CC12.3 has been observed not to form it. Notably, an interesting feature of CDR H3 region of IGHV3-53-encoded antibodies is its short length. Accordingly, CC12.1 and CC12.3 have a CDR H3 consisting of nine amino acids in lengths. This structural feature may rely on the fact that the epitopes of IGHV3-53 antibodies are relatively flat and present a small pocket to insert the CDR H3 loop. Hence, longer CDR H3 regions might not be accommodated in IGHV3-53-encoded antibodies.

**Fig. 1:**
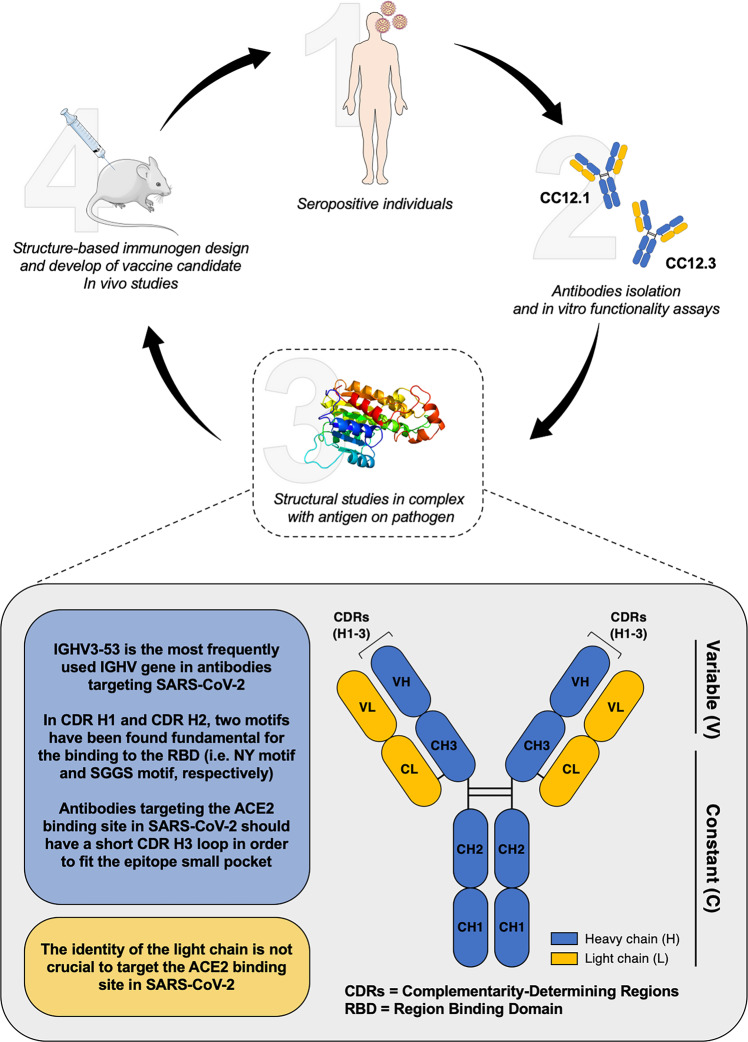
Representation of “reverse vaccinology 2.0” theory: focus on the molecular features of IGHV3-53-encoded antibodies. Monoclonal antibodies are obtained from seropositive subjects, isolated and structurally characterized. Based on the molecular features, a structure-based immunogen is designed and then tested in appropriate animal models

In sum, based on this structural characterization, Yuan and collaborators shed lights on some key molecular features (illustrated in Fig. 1) contributing to an effective antibody response against SARS-CoV-2 infection, demonstrating that IGHV3-53 provides a versatile framework to target the ACE2 binding site in SARS-CoV-2 RBD.

In conclusion, understanding of IGHV3-53-encoded antibodies and, in general, of anti-SARS-CoV-2 neutralizing antibodies, produced by infected donors, is required to generate immunogens that optimally present neutralizing epitopes to the immune system. Such approach may further open new horizons toward the identification of multiple functional antibodies, derived from several donors and directed toward single epitopes regions in order to combine sites of different shapes recognizing the critical regions, thereby capturing the biological diversity of antibody response.^2^ The characterization by Yuan et al. may also allow to create anti-SARS-CoV-2 antibody templates for immunogens design, thus greatly improving the sophistication in the design of immunogens and in immunization strategies.
